# Perceived Partner Responsiveness and Depression: The Moderating Role of Age and Gender

**DOI:** 10.1093/geroni/igab046.3514

**Published:** 2021-12-17

**Authors:** Betul Urganci

**Affiliations:** Cornell University, Ithaca, New York, United States

## Abstract

A growing body of research suggests that greater perceived partner responsiveness- the extent to which individuals feel cared for, understood, and validated by their romantic partner- leads to longer, healthier, and happier life in adulthood, yet little is known about possible moderating factors between responsiveness and well-being. Using a longitudinal design, the current study tested the moderating roles of age and gender in association between perceived partner responsiveness and depression symptoms. The data for the present study came from the National Survey of Midlife in the United States (MIDUS I and II) which is a longitudinal study on health and aging. A life span sample of 2856 married or cohabiting individuals (1402 Female, Mage= 47.16) completed measures of perceived partner responsiveness, depression, age, and gender in two waves (T1 and T2). The results showed that greater perceived partner responsiveness at T1 predicted lower depressive symptoms at T2 controlling for depressive symptoms at T1. This finding remained when controlling for potential confounders including demographics and health covariates. The moderation analysis demonstrated that participants’ age was not a significant moderator in the association between perceived partner responsiveness and depression. Yet, gender significantly was a significant moderator such that the association of perceived partner responsiveness and depression was significant for female but not for male participants. These findings can have implications for mental health and relational well-being.

